# Probiotics Attenuate Myopathic Changes in Aging Rats via Activation of the Myogenic Stellate Cells

**DOI:** 10.1007/s12602-023-10202-2

**Published:** 2023-12-19

**Authors:** Nehal H. M. Abdel-Halim, Eman A. E. Farrag, Maha O. Hammad, Ola Ali Habotta, Hend M. Hassan

**Affiliations:** 1 Physiology Department, Faculty of Medicine, Mansoura University, Mansoura, 35511 Egypt; 2 Clinical Pharmacology Department, Faculty of Medicine, Mansoura University, Mansoura, 35511 Egypt; 3 Medical Biochemistry and Molecular Biology Department, Faculty of Medicine, Mansoura University, Mansoura, 35511 Egypt; 4 Forensic and Toxicology Department, Faculty of Veterinary Medicine, Mansoura University, Mansoura, 35511 Egypt; 5 Human Anatomy and Embryology Department, Faculty of Medicine, Mansoura University, Mansoura, 35511 Egypt

**Keywords:** Aging, Myopathy, Stellate cells, Probiotics, Myogenin, CD34

## Abstract

Aging represents a complex biological process associated with decline in skeletal muscle functions. Aging impairs satellite cells that serve as muscle progenitor cells. Probiotic supplementation may have many beneficial effects via various mechanisms. We examined the possible effects of probiotics in stimulating the proliferation of myogenic stellate cells in aging rats**.** Twenty-four male albino Sprague–Dawley rats were classified equally into four groups: adult control, old control, adult + probiotics, and old + probiotics. Probiotics (Lactobacillus LB) were administered gavage at a dose of 1 ml (1 × 10^9^ CFU/ml/day) for 4 weeks. A significant increase in the relative gastrocnemius weight ratio and improvement of contractile parameters was detected in the old + probiotics group (0.6 ± 0.01) compared to the old control group (0.47 ± 0.01; *P* < 0.001). Probiotics significantly upregulated the activities of GSH, while NO and MDA were markedly decreased compared to control groups (*P* ≤ 0.001). Also, probiotics increased the mRNA and protein expressions of myogenin and CD34 (*P* < 0.05) as determined by real-time PCR and immunohistochemistry. Moreover, the old + probiotics group showed apparent restoration of the connective tissue spaces, reflecting the all-beneficial effects of probiotics. Our findings indicated that probiotics attenuated myopathic changes in aging rats probably through activation of the myogenic stellate cells. Probiotics improved the muscle weight, function, antioxidant activity, and myogenic transcription factors of the skeletal muscle.

## Introduction

Aging is an inevitable and complex biological process that is characterized by a deterioration in the physiological and biochemical activities of the major systems [1]. Since skeletal muscle is the largest organ in the body, sarcopenia, the age-related loss of skeletal muscle volume and strength, seems to be an unavoidable aspect of aging [2]. Between the ages of 60 and 70, the prevalence increases by 5–13%, and above the age of 80, it increases by 11–50%. The prevalence is expected to climb by 30% in the population over 60 years old by 2050, dramatically increasing the number of sarcopenia patients [3]. This disease is characterized by an increased risk of falls and fractures due to a decline in muscle bulk and strength and limited mobility. Ongoing research on sarcopenia principally focuses on improving the disease and decreasing the economic burden of this grave condition in the elderly [4].

There are several mechanisms involved in aging and skeletal muscle changes. Oxidative impairment has been anticipated as one of the major reasons for the skeletal muscle weakening with aging [5]. Moreover, changed hormonal status, chronic inflammation, α-motor neuron loss, muscular mitochondrial damage, myocyte autophagy alteration, myonuclei apoptosis acceleration, and satellite cell impairment are believed to be the most important factors and contributors to age-related muscle wasting [6].

Satellite cells, small quiescent cells, are placed between the basal lamina and myofiber membrane and work as muscle progenitor cells for muscle growth and repair [7]. Satellite cells have been recognized and isolated based on their expression of CD34, a sialo-mucin surface receptor conventionally used as a marker of hematopoietic stem cells [8].

Satellite cell stimulation resulted in proliferation and differentiation directed by the general cell cycle regulatory machinery and myogenic regulatory transcription factors (MRFs), which include MyoD, Myf5, and Myogenin [9]. With aging, muscle satellite cells are largely impacted by environmental signals, leading to a decline in their population and a deficiency of their activity that disturbs muscle regeneration. Aging harms both autonomous and niche-dependent regulation of muscle satellite cells, causing alterations in their capacity to maintain quiescence and functional loss of self-renewal and regeneration, the leading causes driving age-linked muscle alterations [10].

The gut microbiota (probiotics) plays a significant role in the host’s nutrition, metabolism, immunity, and neurological functions [11]. Imbalanced microbiota is related to stunting, inflammatory, metabolic, muscular disorders, and cancer where this imbalance triggers immunity and systemic inflammation, which leads to a variety of illnesses [11, 12]. Probiotic supplementation may have many beneficial effects through numerous mechanisms of action, though the exact mechanism by which they exert their effects has not yet been fully clarified [13].

Many reviews linked the gut microbiota with muscle physiology, metabolism, and function through the “gut-muscle axis” and how this axis may be involved in the onset and clinical course of sarcopenia either directly or indirectly [12, 14]; however, the mechanisms of probiotics are complex and often poorly understood. Thus, we hypothesized that probiotic supplementation may alleviate aging-related sarcopenia through modulation of the activity of the myogenic stellate cells and its antioxidant effect.

This study is intended to examine the potential effects of probiotics on the biochemical, immunohistochemical, and molecular changes associated with aging-associated myopathy and the possible impacts of probiotics in stimulating the proliferation of myogenic stellate cells in aging rats, aiming to alleviate the aging-associated myopathic changes.

## Materials and Methods

### Animals and Ethical Approval

This study was conducted on twenty-four male Sprague–Dawley albino rats. Adult rats are aged 12–16 weeks, while old rats are aged 22–24 months. The rats used in this study weighed 200–400 g and were purchased from Mansoura medical experimental center. They were preserved under ordinary environmental conditions of a 12:12-h dark–light cycle, a constant temperature of 22 ± 3 °C, and a relative humidity of 50 ± 10%. A well-adjusted standard diet and water were freely provided through the experiment. Rats were adapted for 2 weeks preceding the beginning of the study. The research procedure was revised and approved by the Committee of Research Ethics for Laboratory Animal Care (code no. R/114), Faculty of Veterinary Medicine, Mansoura University, Egypt.

### Experimental Design

Rats were randomly separated into four groups (six rats each) as follows: (I) adult control group, adult rats fed on an ordinary rodent chow; (II) old control group, aging rats fed on an ordinary rodent chow; (III) adult + probiotic group, adult rats supplemented with probiotic (Lactobacillus LB sachet; 10 billion, manufactured by RAMED under license of ADARE Pharmaceuticals SAS-France) orally by gastric gavage at a dose of 1 ml (1 × 10^9^ CFU/ml/day) [15] for 4 weeks; (IV) old + probiotic group, aging rats supplemented with probiotic (*Lactobacillus acidophilus*) orally by gastric gavage at a dose of 1 ml (1 × 10^9^ CFU/ml/day) for 4 weeks.

### Specimens’ Collection

A day after the last dose of the medication, the rats were euthanized with 300 mg/kg of intraperitoneal chloral hydrate. Gastrocnemius muscles were dissected out carefully. One muscle was used to measure the contractile parameters by computerized data acquisition system unit MP45 (BIOPAC Student Lab 3.7.3.). The other one was divided into three portions: the first was homogenized in 0.05 M ice-cold phosphate buffer (pH; 7.5) using a tissue homogenizer at 40 °C, then centrifuged at 10,000 rpm for 20 min, and the supernatant was collected and stored on ice until conducting the biochemical analysis; the second was collected in RNAlater (10 µl RNAlater per 1 mg tissue) (Qiagen, Germany), placed at 4 °C for 1 day, and then stored at − 80 °C for analysis of gene expression; and the last one was fixed in 10% neutral buffered formalin to perform the histopathological and immunohistochemical analysis.

### Calculation of the Relative Weight of Gastrocnemius Muscle

A precise balance scale measured the muscle wet weight. The muscle-relative weight ratio was calculated as the ratio of the wet weight of the muscle divided by the body weight at the finish of the experiment according to the following formula [16].

$$\mathrm{Relative}\;\mathrm{weigt}\;\mathrm{ratio}\;\mathrm{of}\;\mathrm{the}\;\mathrm{muscle}=\frac{\mathrm{wet\;weight\;of\;the\;muscle}}{\mathrm{body\;weight\;of\;the\;rat}}\times100\%$$

### Recording of the Contractile Parameters by BIOPAC

Gastrocnemius was dipped in Krebs solution at 30 °C. The Krebs solution had the following constituents (mM): 120 NaCl; 5.0 KCI; 25 NaHCO_3_; 1.2 NaH_2_PO_4_; 2.5 calcium gluconate; 1.2 MgSO_4_; 11.5 glucose. It was constantly bubbled with a mix of 5% C0_2_ and 95% O_2_, and the PH was maintained at 7.4. The biopac apparatus was adjusted by connecting the BSLSTM stimulator to channel one (1), and recording electrode (SS12LA) was connected to channel two (2). On the muscle, the stimulating electrodes were positioned for direct stimulus. Channels were set up and modified the baseline. At first, maximal isometric twitch was recorded by applying single maximal stimulation (30 V) [17]. Then, the tetanic contraction was recorded by providing a continuous stimulation. The tetanic frequency in gastrocnemius was 60 HZ [18]. The contractile parameters were measured: maximum isometric twitch force (Pt), maximum tetanic force (P0), maximum specific isometric tetanic force (SP0), half relaxation time (1⁄2RT), and time to peak (TP).The maximum specific force (SP0), cross-sectional area (CSA), and equal maximum tetanic force per cross-sectional area were estimated by scaling histopathological specimens [19].

### Evaluating Oxidative Stress Biomarkers in the Gastrocnemius Homogenate

Oxidative stress status was evaluated by assessing reduced glutathione (GSH), malondialdehyde (MDA), and nitric oxide (NO) content in muscle tissue homogenates using colorimetrically available kits (Biodiagnostic, Egypt) and following the manufacturers’ guidelines. The GSH was evaluated according to the method of Beutler et al. [20]. The method is established on the development of a yellow color when GSH reacts with 5,5′dithiobis (dithionitrobenzoic acid) (DTNB) via its sulfhydryl group. The absorbance of the yellow chromogen was measured at 405 nm. MDA level was detected as an indicator of lipid peroxidation by the Ohkawa et al. method [21] in which MDA was coupled with thiobarbituric acid (TBA), and the resultant pink MDA-TBA products were calculated at 535 nm spectrophotometrically. NO is colorimetrically measured based on the method of Montgomery and Dymock [22]. In acidic media and in the presence of nitrite, the produced nitrous acid diazotize sulphanilamide and the product reacted with N-(1–naphthyl) ethylenediamine. The resultant azo dye showed a violet color which could be read calorimetrically at 540 nm.

### Myogenin and CD34 mRNA Quantification by Real-time PCR Analysis

Myogenin and CD34 mRNA expressions were evaluated by reverse transcription-polymerase chain reaction (RT-PCR). Total muscle RNA was extracted and purified with the QIAzol reagent (Qiagen, Germany) according to the manufacturer’s protocol. The purity of RNA was checked using NanoDrop by obtaining the optical density (OD) 260/280 ratios (Thermo Fisher Scientific, USA). RNA (1 µg per sample) was used to synthesize complementary DNA (cDNA) using the SensiFAST™ cDNA Synthesis Kit according to the manufacturer’s guidelines (Bioline, UK). Reverse-transcribed cDNA templates were amplified using real-time PCR apparatus (Applied Biosystems 7500, USA) with primers specific for myogenin and CD34.

Primers were designed using Primer3 software (v.4.1.0; http://primer3.ut.ee) for the analysis of the expression of myogenin mRNA, CD34 mRNA, and GAPDH mRNA. GAPDH was used as the ubiquitous control gene. The myogenin primers (*Rattus norvegicus*; PCR amplicon: 103 bp; RefSeq: NM_017115.3): F:5′-CGGTGGTACCCAGTGAATGC-3′ and R:5′-GCTGCGAGCAAATGATCTCC-3′; CD34 primers (*Rattus norvegicus*; PCR amplicon: 145 bp; RefSeq: NM_001107202.2) F:5′-GGAGCCACCAGAGCTATTCC-3′ and R:5′-TAAGGGTCTTCACCCAGCCT-3′; and the GAPDH primers (*Rattus norvegicus*; PCR amplicon: 169 bp; RefSeq: NM_017008.4) F: 5′- CCTCGTCTCATAGACAAGATGGT -3′ and R:5′-GGGTAGAGTCATACTGGAACATG -3′. The PCR reaction was performed using 10 µl of HERA SYBR green PCR Master Mix (Willowfort, UK), 2 µl of cDNA, applying 2 µl (10 pmol/µl) primer and 6 µl of nuclease-free water to 20 µL volume. Thermal cycling conditions included initial denaturation of 2 min at 98 °C, followed by 40 cycles of 10 s at 95 °C, and 30 s at 60 °C.

The amplification plots of all real-time PCR products were analyzed for obtaining the cycle threshold (Ct) of myogenin, CD34, and GAPDH. The fold changes in the target, and housekeeping genes for the results of relative quantitation were calculated for each sample using the 2^−∆∆Ct^ method. ∆Ct is the difference between the Ct of the target gene and the Ct of the GAPDH housekeeping gene in the same sample. ∆∆Ct represents the difference between the mean ∆Ct value of a diseased sample and the mean ∆Ct of the control sample (calibrator), both calculated after the same PCR run [23].

### Histopathological Examination of Gastrocnemius Tissue Specimens

Preserved gastrocnemius tissues were subjected to ordinary histopathological steps as they were dehydrated in graded alcohol and xylene, embedded in paraffin wax, followed by sectioning by a slicer. The tissue sections (4–5 µm) were stained with H&E to evaluate the pathological changes. Also, muscle sections were stained with Masson trichrome stain for assessment of interstitial fibrosis and collagen deposition, which appeared bluish in color.

### Immunohistochemical Expression of CD34 and Myogenin in Gastrocnemius Tissue Specimens

In order to stop endogenous peroxidases, 0.03% H_2_O_2_ was utilized. The antigens were first prepared in buffered saline containing 5% bovine serum albumin for 20 min in a microwave with sodium citrate buffer (pH = 6). Sections were then treated for a whole night at 4 °C with a primary antibody against CD34 (1:2500, ab81289) and myogenin (1:500, ab124800). The avidin-biotinylated peroxidase complex (ABC-kit) and DAB substrate (ab64238) were used in accordance with the manufacturer’s instructions to detect the reaction. After slices were dehydrated in progressively stronger alcohols, cleaned in xylene, and mounted, hematoxylin was used as a counterstain [24].

### Computer-Assisted Digital Image Analysis (Digital Morphometric Study) for Measurement of the area % of Positive Myogenin and CD34 Immune Reaction

Each rat in each group had five randomly placed sections investigated. Using a 20 objective, the area fraction of immunological expression was detected. Using the Image-j computerized image analysis system (version 1.48), the immune-positive reaction was examined. Almost all of each section’s layers displayed a distinctive brownish color that was immune expression. The color content of each image was separated using the color deconvolution plugin. For more accuracy, the threshold was modified [25].

### Statistical Analysis

Data were displayed as the mean ± standard error of mean (SEM) and analyzed by one-way analysis of variance (one-way ANOVA), followed by the Games-Howell post hoc test, using the statistical package SPSS version 26.0. *P*-values ≤ 0.05 were considered statistically significant.

## Results

### Effect of Probiotics on Relative Gastrocnemius Weight Ratio Measurement

The old control group’s muscle weight ratio significantly decreased when compared to the adult control group (*P* ≤ 0.001). There was no significance between adult + probiotics group and adult control group (*P* = 0.2). However, the old + probiotics group showed a significant increase in muscle weight ratio compared to probiotic-free old group (*P* < 0.001). Moreover, there was a significant reduction in old + probiotics group compared to the adult + probiotics group (*P* < 0.004) (Table 1).

**Table 1:** Relative muscle weight ratio measurement results between the study groups

	**Adult control (*N* = 6)**	**Old control (*N* = 6)**	**Adult + probiotics (*N* = 6)**	**Old + probiotics (*N* = 6)**	***P*-values**
Weight ratio	0.66 ± 0.01	0.47 ± 0.01^a^	0.7 ± 0.01^b^	0.6 ± 0.01 ^c,d^	≤ 0.001*

Data were displayed by mean ± SEM. The four groups were compared via one-way ANOVA followed by post hoc Games-Howell test for pairwise comparison

^*^P-value among all studied groups

^a^Comparison between old control and adult control groups, < 0.001

^b^Comparison between adult + probiotics and adult control groups, 0.2

^c^Comparison between old + probiotics and old control groups, < 0.001

^d^Comparison between old + probiotics and adult + probiotics groups, 0.004

### Effect of Probiotics on Biopac Results

To evaluate the effect of probiotics on the improvement of gastrocnemius muscle function, the contractile parameters were assessed in the four different groups using Biopac. The old control group displayed a marked decrease in Pt, P0, and SP0 compared to the adult control group (Pt ≤ 0.001, P0 ≤ 0.001, SP0 ≤ 0.001). P0 and SP0 significantly increased in the adult + probiotics group compared to the adult control group (P0 ≤ 0.001, SP0 ≤ 0.001); however, there was no significance between the two groups in Pt (*P* < 0.8). Probiotic-treated old group exhibited a significant rise in Pt, P0, and SP0 (Pt ≤ 0.001, P0 ≤ 0.001, SP0 ≤ 0.001) compared to probiotic-free old group (*P* ≤ 0.001). Compared to the adult + probiotics group, the old + probiotics group exhibited a significant decline in Pt, P0, and SP0 (Pt ≤ 0.001, P0 ≤ 0.001, SP0 ≤ 0.001). No changes were noticed with regard to TP in all groups (Table 2).

**Table 2:** Biopac results between the study groups

	**Adult control (*N* = 6)**	**Old control (*N* = 6)**	**Adult + probiotics (*N* = 6)**	**Old + probiotics (*N* = 6)**	***P*-values**
Pt	12.28 ± 0.3	1.59 ± 0.08 ^a^	12.6 ± 0.3 ^b^	5.57 ± 0.4 ^c,d^	< 0.001*
P0	17.27 ± 0.7	3.13 ± 0.12 ^a^	20.11 ± 0.5 ^b^	10.76 ± 0.9 ^c,d^	< 0.001*
SP0	11.5 ± 0.4	2.08 ± 0.08 ^a^	13.4 ± 0.34 ^b^	7.17 ± 0.6 ^c,d^	< 0.001*
TP	0.05 ± 0.01	0.05 ± 0.01	0.05 ± 0.01	0.05 ± 0.01	1
1\2RT	0.04 ± 0.01	0.05 ± 0.01	0.04 ± 0.01	0.05 ± 0.01	< 0.001*

Data were displayed by mean ± SEM. The four groups were compared via one-way ANOVA followed by post hoc Games-Howell test for pairwise comparison

^a^Comparison between old control and adult control groups: (Pt ≤ 0.001, P0 ≤ 0.001, SP0 ≤ 0.001)

^b^Comparison between adult + probiotics and adult control groups: (Pt 0.8, P0 = 0.02, SP0 = 0.02)

^c^Comparison between old + probiotics and old control groups: (Pt ≤ 0.001, P0 ≤ 0.001, SP0 ≤ 0.001)

^d^Comparison between old + probiotics and adult + probiotics groups: (Pt ≤ 0.001, P0 ≤ 0.001, SP0 ≤ 0.001)

Abbreviations: *Pt* maximum isometric twitch force, *P0* maximum tetanic force, *SP0* maximum specific isometric tetanic force, *TP* time to peak, *1/2RT* half relaxation time

### Effect of Probiotics on Oxidative Stress Markers in Gastrocnemius Muscle Homogenate

To study the effect of probiotics on the improvement of muscle function in aging rats, we examined the oxidative stress markers. Aging caused muscle oxidative stress as indicated by increased levels of NO and MDA and decreased level of GSH in the old control rats compared to the adult control rats (*P* ≤ 0.001). Probiotics exhibited an antioxidant ability by significantly reverse this change in the adult + probiotics group when compared to adult controls (*P* ≤ 0.001) and the old + probiotics group when compared to the old controls (*P* ≤ 0.001). However, the adult + probiotics and old + probiotics groups did not differ statistically significantly in terms of NO (*P* = 0.5), MDA (*P* = 0.16), and GSH (*P* = 0.5) (Table 3).

**Table 3:** Comparison of the levels of oxidative stress markers in rat gastrocnemius muscle homogenate between the study groups

	**Adult control (*N* = 6)**	**Old control (*N* = 6)**	**Adult + probiotics (*N* = 6)**	**Old + probiotics (*N* = 6)**	***P*-values**
NO (µmol/l)	2.15 ± 0.03	4.5 ± 0.1 ^a^	3.21 ± 0.1 ^b^	3.39 ± 0.08 ^c,d^	< 0.001*
MDA (nmol/g)	12.1 ± 0.4	22.5 ± 0.3^a^	17.5 ± 0.5 ^b^	18.85 ± 0.25 ^c,d^	< 0.001*
GSH (mmol/mg)	1.76 ± 0.03	0.83 ± 0.03^a^	3.22 ± 0.1 ^b^	3.39 ± 0.09^c,d^	< 0.001*

Data are displayed by mean ± SEM. The four groups were compared via one-way ANOVA followed by post hoc Games-Howell test for pairwise comparison

^a^Comparison between the old control and the adult control groups, *P* ≤ 0.001

^b^Comparison between the adult + probiotics and the adult control groups, *P* ≤ 0.001

^c^Comparison between the old + probiotics and the old control groups, *P* ≤ 0.001

^d^Comparison between the old + probiotics and the adult + probiotics groups: (NO, *P* = 0.5; MDA, *P* = 0.16; GSH, *P* = 0.5)

Abbreviations: *NO* nitric oxide, *MDA* malondialdehyde, *GSH* reduced glutathione

### Effect of Probiotics on Myogenin and CD34 mRNA Levels

**Fig. 1:**
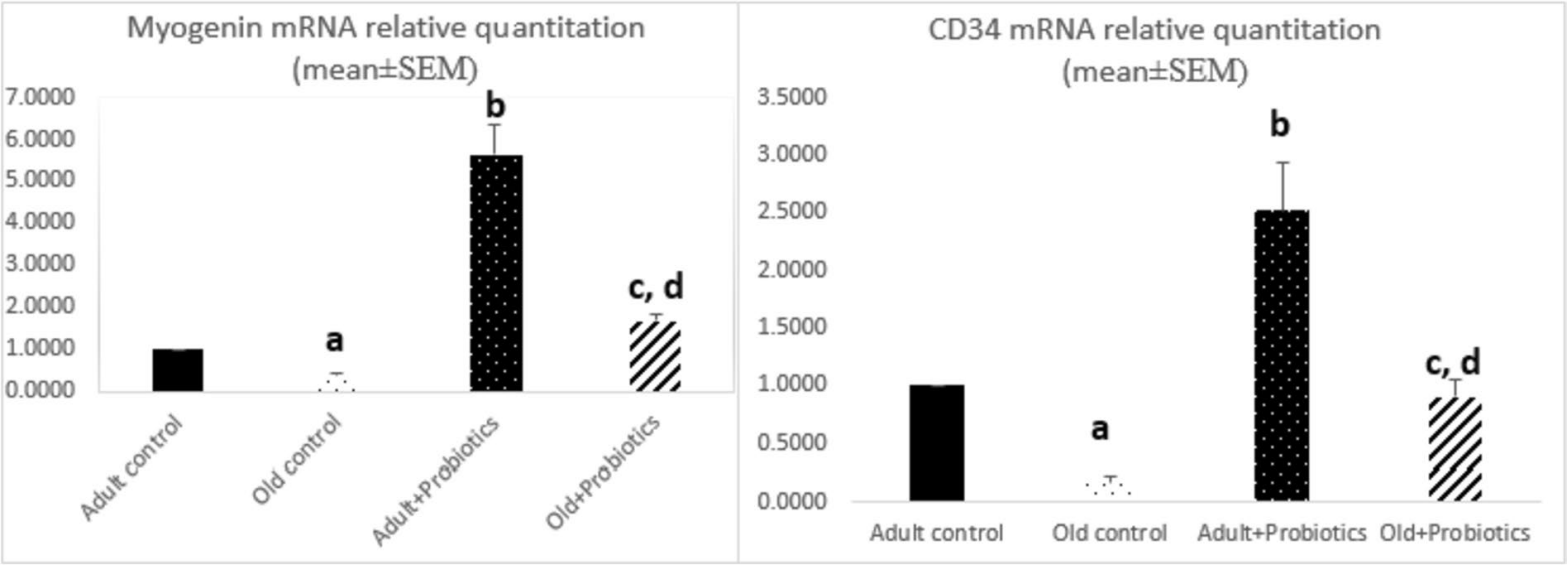
Evaluation of myogenin and CD34 mRNA levels in rat gastrocnemius muscle tissues among the study groups. Data were displayed by mean ± SEM. The four groups were compared via one-way ANOVA followed by post hoc Games-Howell test for pairwise comparison. a, comparison between the old control and the adult control groups, *P* ≤ 0.001; b, comparison between the adult + probiotics and the adult control groups (Myogenin *P* ≤ 0.001; CD34, *P* = 0.02); c, comparison between the old + probiotics and the old control groups (Myogenin *P* ≤ 0.001; CD34, *P* = 0.008); d, comparison between the old + probiotics and the adult + probiotics groups (Myogenin *P* = 0.003; CD34, *P* = 0.02)

In order to assess how the probiotics affected the gene expression of myogenin and CD34 in aging rats, their mRNA levels were assessed in rat gastrocnemius muscle tissues via qRT-PCR (Fig. 1). Aging decreased the myogenin mRNA level in the old control rats compared to the adult controls (*P* ≤ 0.001). Our results reported that probiotics significantly increased the myogenin mRNA level in the adult + probiotics group compared to either the adult control rats (*P* ≤ 0.001) or the old + probiotics group (*P* = 0.003). Moreover, the probiotic supplementation produced a significant rise in myogenin gene expression in the old + probiotics group compared to the old controls (*P* ≤ 0.001). Regarding CD34 gene expression, the old controls revealed a marked decrease in the CD34 mRNA level compared to the adult controls (*P* ≤ 0.001) and the old + probiotics group (*P* = 0.008). Probiotic supplementation to the adult rats produced a significant increase in CD34 mRNA expression compared to the adult controls (*P* = 0.02), old controls (*P* ≤ 0.001), or the old + probiotics group (*P* = 0.02) (Fig. 1).

### Effect of Probiotics on Immunohistochemical Analysis of Myogenin and CD34

**Fig. 2:**
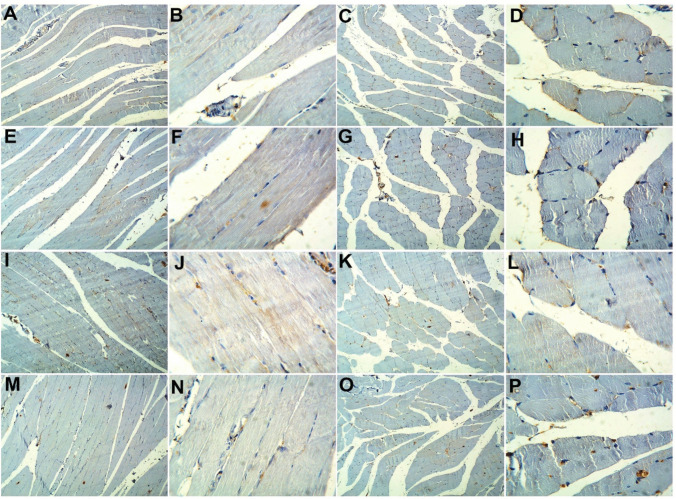
Immunohistochemical evaluation of myogenin in rat gastrocnemius muscle tissues among the study groups. The adult control group showed positive immunoreactivity in both longitudinal (**A** and **B**) and transverse sections (**C** and **D**). The old control group showed apparent decreased reactivity in either the longitudinal (**E** and **F**) or transverse sections (**G** and **H**). The adult + probiotics group showed relative increased reactivity in the longitudinal (**I** and **J**) and transverse sections (**K** and **L**). The old + probiotics group showed an apparent increased reaction in the longitudinal (**M** and **N**) and transverse sections (**O** and **P**). (**A**, **C**, **E**, **G**, **I**, **K**, **M**, and **O**, X100; **B**, **D**, **F**, **H**, **J**, **L**, **N** and **P**, X 400)

For further confirmation of myogenin and CD34 RT-PCR results, immunohistochemical evaluation of myogenin and CD34 in rat gastrocnemius muscle tissues was performed. Myogenin immune staining of sections of gastrocnemius muscle in the adult control group exhibited a positive immunoreactivity in both longitudinal and transverse sections. The old control group showed apparent decreased reactivity in either the longitudinal or transverse sections. The adult + probiotics group showed relative increased reactivity in the longitudinal and transverse sections. The old + probiotics group showed an apparent increased reaction in the longitudinal and transverse sections (Fig. 2).

**Fig. 3:**
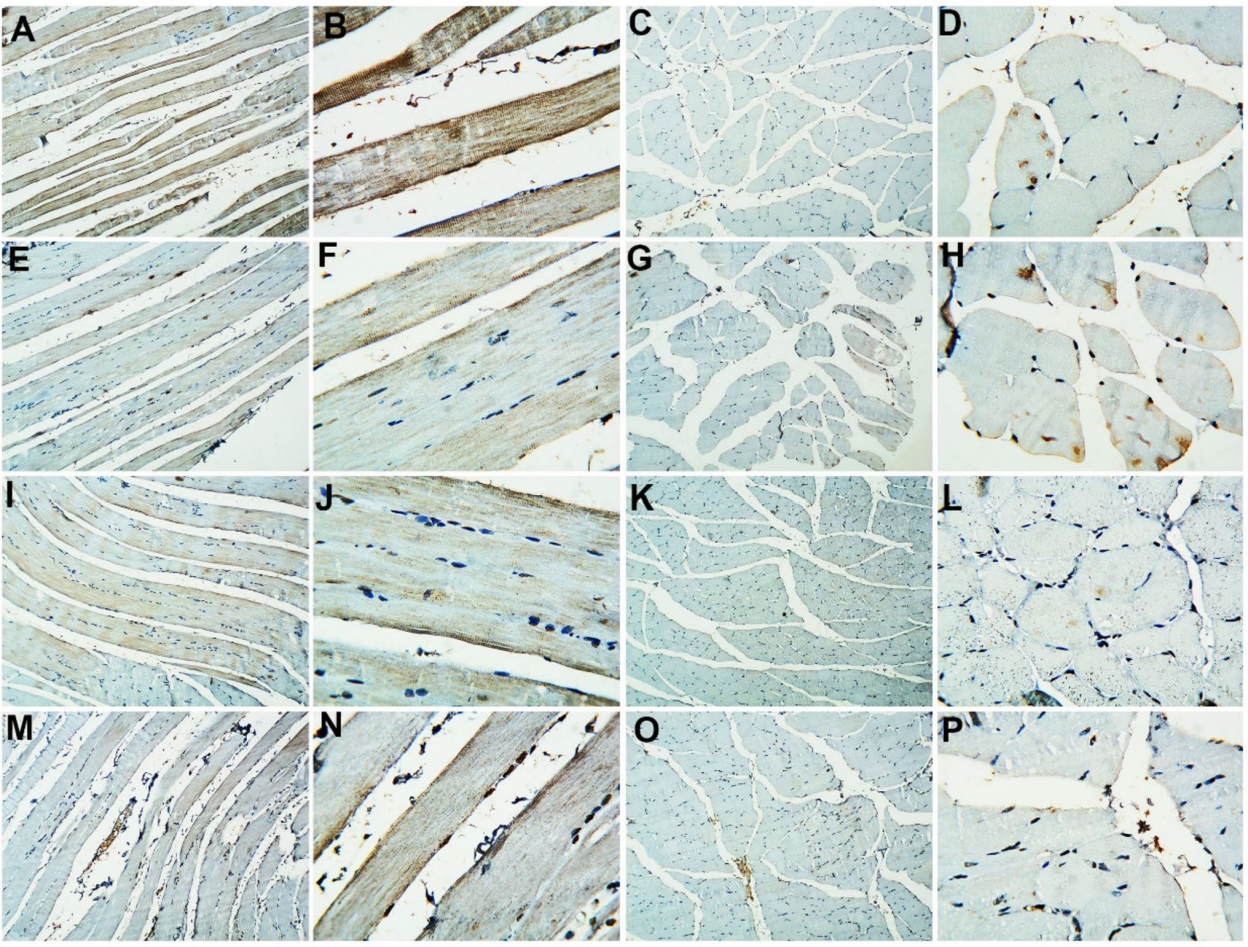
Immunohistochemical evaluation of CD34 in rat gastrocnemius muscle tissues among the study groups. The adult control group showed a positive immunoreactivity in both longitudinal (**A** and **B**) and transverse sections (**C** and **D**). The old control group showed an apparent decreased reactivity in either the longitudinal (**E** and **F**) or transverse sections (**G** and **H**). The adult + probiotics group showed relative increased reactivity in the longitudinal (**I** and **J**) and transverse sections (**K** and **L**). The old + probiotics group showed more or less similar reaction in the longitudinal (**M** and **N**) and transverse sections (**O** and **P**). (**A**, **C**, **E**, **G**, **I**, **K**, **M**, and **O**, X100; **B**, **D**, **F**, **H**, **J**, **L**, **N**, and **P**, X 400)

CD34 immune staining of sections of the gastrocnemius of the adult control group exhibited positive immunoreactivity in both longitudinal and transverse sections. The old control group showed apparent decreased reactivity in either the longitudinal or transverse sections. The adult + probiotics group showed relative increased reactivity in the longitudinal and transverse sections. The old + probiotics group showed more or less similar reaction in the longitudinal and transverse sections (Fig. 3).

### Morphometric Results of Myogenin and CD34 Area% of Immune Reaction Measurement

Regarding the area percentage of myogenin and CD34 in different groups, aging decreased the myogenin level in the old controls when compared to the adult controls (*P* = 0.03). Probiotics significantly increased the myogenin level in the adult + probiotics group compared to the adult control rats (*P* < 0.03). Moreover, the probiotics produced a significant increase in myogenin level in the old + probiotics group compared to the old control rats (*P* = 0.003). However, there was a considerable decrease in the old + probiotics group compared to the adult+ probiotics group (*P* ≤ 0.001).

**Fig. 4:**
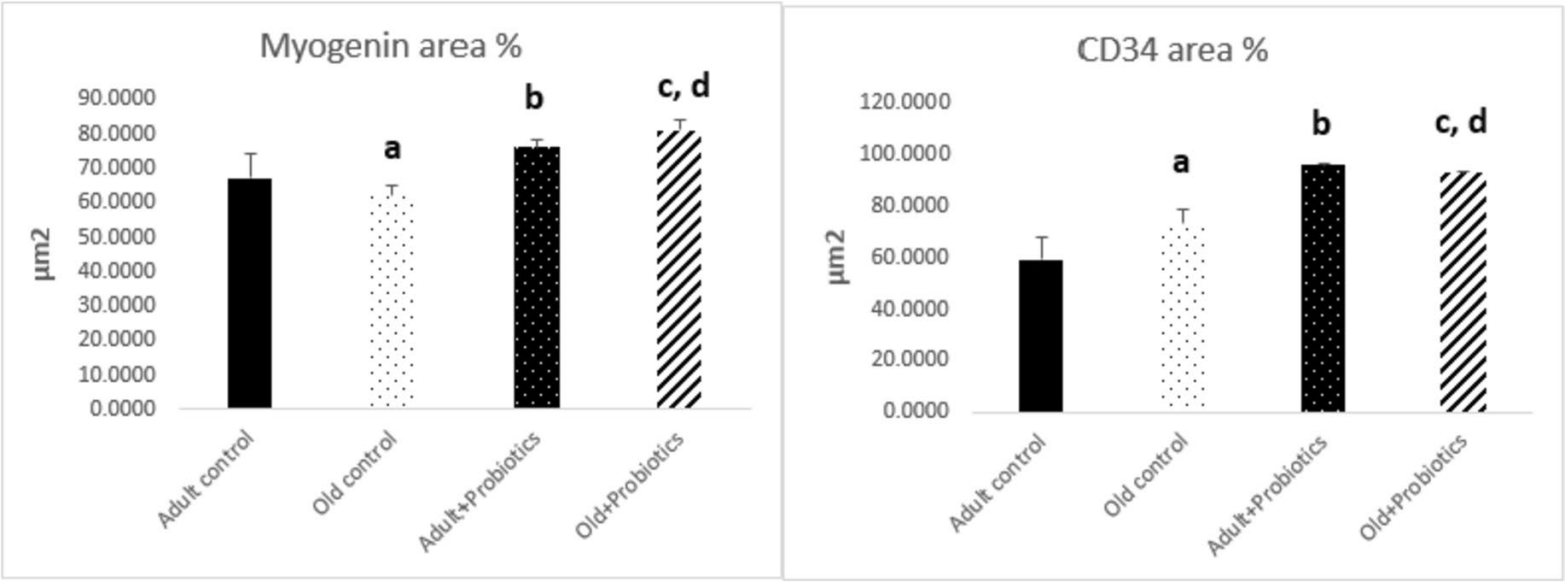
Area percentage of positive myogenin and CD34 levels in rat gastrocnemius muscle tissues among the study groups. Data were displayed by mean ± SEM. The four groups were compared via one-way ANOVA followed by post hoc Games-Howell test for pairwise comparison. *P*-value among all studied groups (myogenin, 0.02; CD34, ≤ 0.001). a, comparison between old control and adult control groups (myogenin, 0.03; CD34, 05). b, comparison between adult + probiotics and adult control groups (myogenin, 0.03; CD34, 0.01). c, comparison between old + probiotics and old control groups (myogenin, 0.003; CD34, 0.05). d, comparison between old + probiotics and adult + probiotics groups (myogenin, ≤ 0.001; CD34, 0.02)

Regarding CD34 level, the old control rats revealed a non-significant rise in the CD34 level compared to the adult control rats (*P* = 0.05). Probiotic supplementation either to adult rats or old rats produced a significant increase in CD34 level compared to either the adult control (*P* = 0.01) or the old control groups (*P* = 0.05) (Fig. 4).

### Gastrocnemius Histopathological Examination Results

**Fig. 5:**
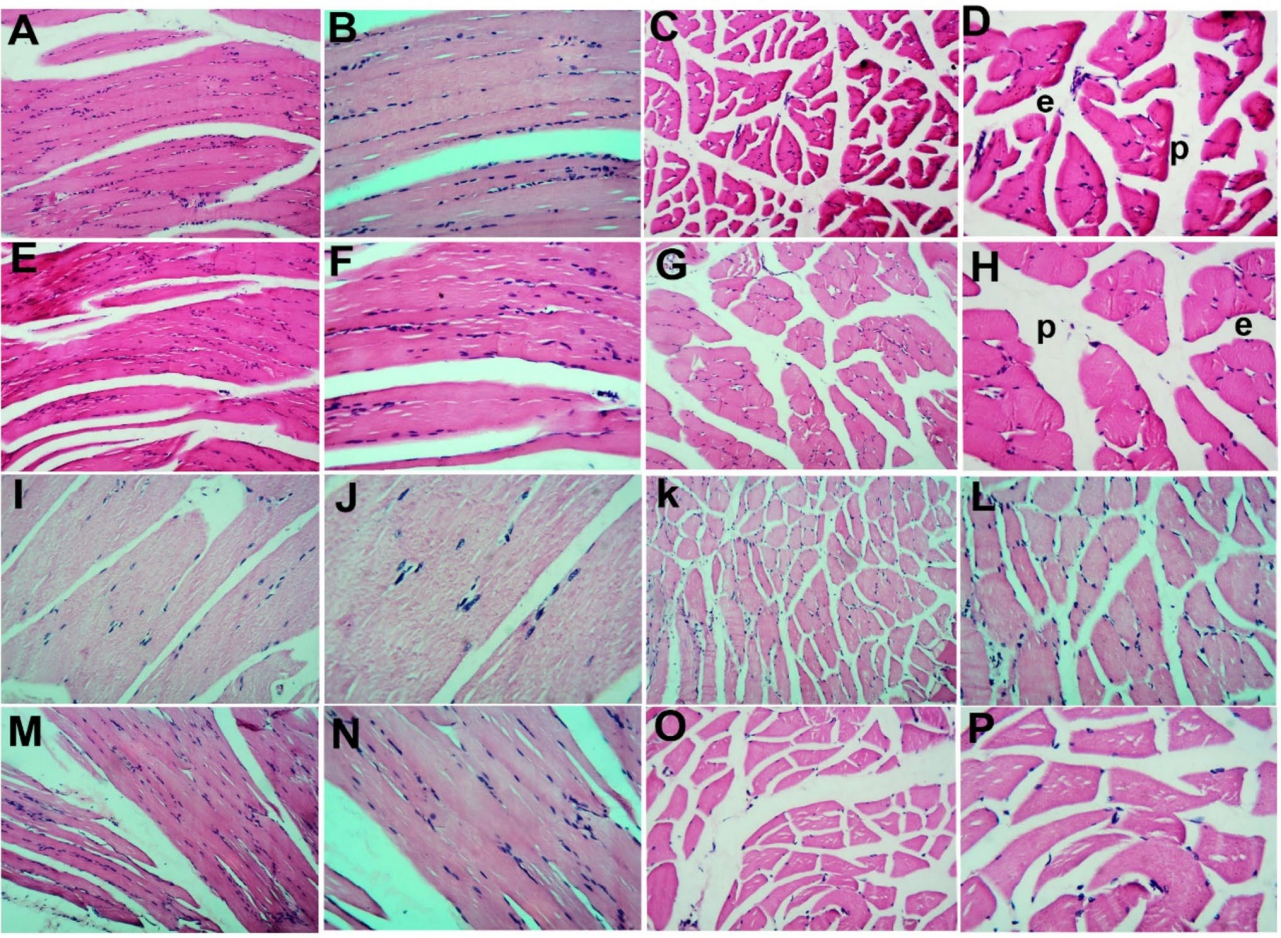
H&E-stained gastrocnemius muscle section. The adult control group exhibited normal distribution and arrangement of the skeletal muscle fibers in the longitudinal sections (**A** and **B**) separated by connective tissue of the perimysium (not stained) and the transverse sections (**C** and **D**) showing polyhedral muscle fibers with acidophilic sarcoplasm and several peripheral oval nuclei separated by narrow C.T. endomysium (e) (not stained) and a wide perimysium between skeletal muscle bundles. The old control group showed apparent increased presentation of the connective tissue spaces of both endo- and perimysia (p and e) in either the longitudinal (**E** and **F**) or transverse sections (**G** and **H**). The adult + probiotics group showed less connective tissue spacing in the longitudinal (**I** and **J**) and transverse sections (**K** and **L**). The old + probiotics group showed more or less similar changes in the longitudinal (**M** and **N**) and transverse sections (**O** and **P**). (**A**, **C**, **E**, **G**, **I**, **K**, **M**, and **O,** X100; **B**, **D**, **F**, **H**, **J**, **L**, **N,** and **P**, X 200)

Hematoxylin and eosin staining of sections of rat gastrocnemius muscle tissues among the study groups were evaluated. The adult control group showed the normal distribution and arrangement of the skeletal muscle fibers in the longitudinal sections divided by the connective tissue of the perimysium and the transverse sections. The muscle fibers were polyhedral with acidophilic sarcoplasm and several oval nuclei in the periphery, separated by a narrow C.T. endomysium and a wide perimysium between skeletal muscle bundles. The old control group showed an apparent increased presentation of the connective tissue spaces of both endo- and perimysia in either the longitudinal or transverse sections. The adult + probiotics group showed less connective tissue spacing in the longitudinal and transverse sections. The old+ probiotics group showed more or less similar changes in the longitudinal and transverse sections (Fig. 5).

**Fig. 6:**
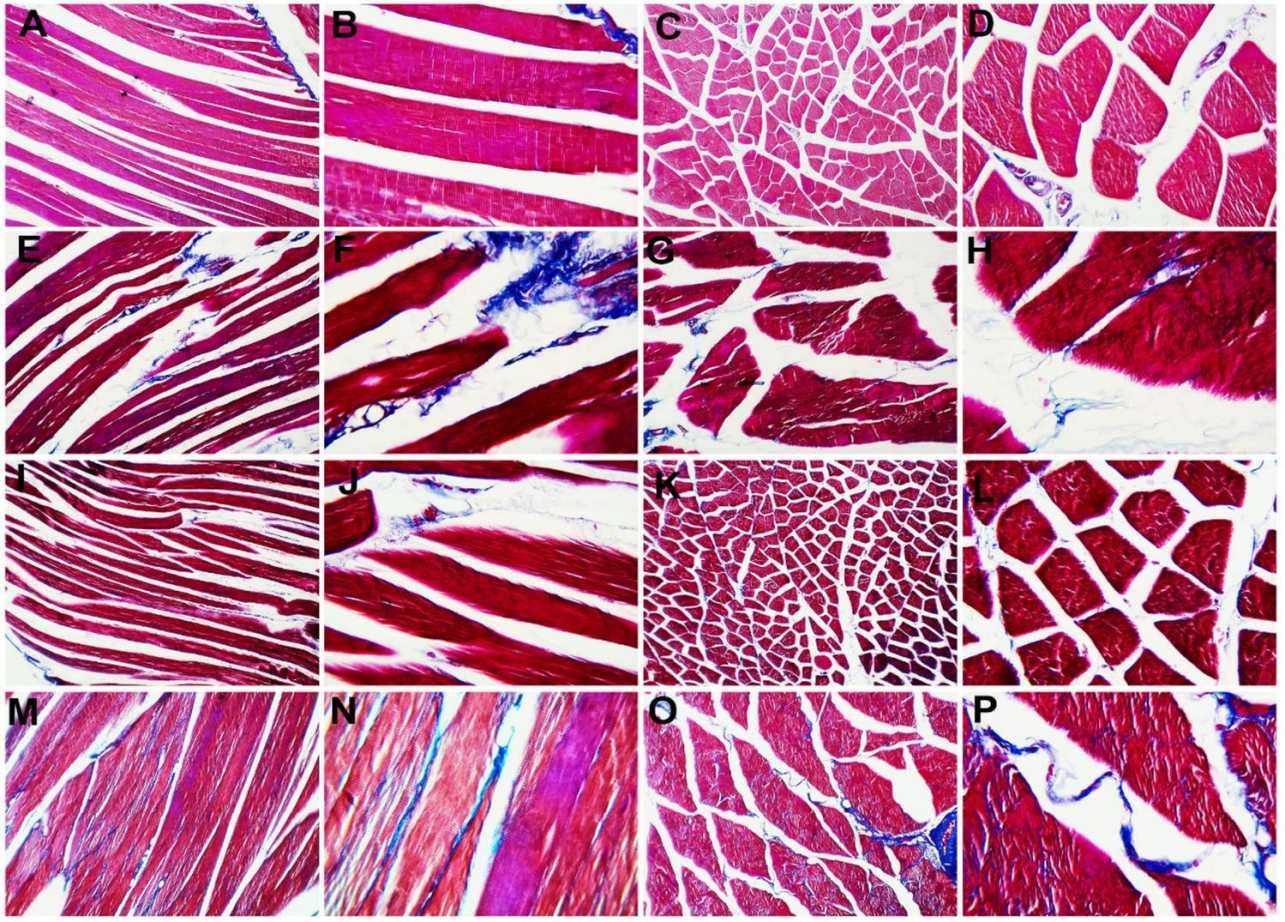
Masson trichrome-stained gastrocnemius muscle section. The adult control group exhibited normal distribution of the bluish-stained collagen fibers in the longitudinal (**A** and **B**) and transverse sections (**C** and **D**). The old control group showed an apparent increased presentation of collagen fibers in the longitudinal (**E** and **F**) and transverse sections (**G** and **H**). The adult + probiotics group showed less marked collagen fibers in the longitudinal (**I** and **J**) and transverse sections (**K** and **L**). The old + probiotics group showed markedly increased collagen fibers in the longitudinal (**M** and **N**) and transverse sections (**O** and **P**). (**A**, **C**, **E**, **G**, **I**, **K**, **M**, and **O** X100; **B**, **D**, **F**, **H**, **J**, **L**, **N,** and **P**, X 400)

Moreover, Masson trichrome staining of sections of rat gastrocnemius muscle tissues was evaluated. The adult control group shows the normal distribution of the bluish-stained collagen fibers in the longitudinal and transverse sections. The old control group showed an apparent increased presentation of collagen fibers in the longitudinal and transverse sections. The adult + probiotics group showed less marked collagen fibers in the longitudinal sections and the transverse sections. The old + probiotics group showed markedly increased collagen fibers in the longitudinal and transverse sections (Fig. 6).

## Discussion

In the current study, we aimed to study the potential role of probiotics in stimulating the proliferation of myogenic stellate cells in aging rats, aiming to alleviate the aging-associated myopathic changes. This study demonstrated for the first time the efficacy of probiotic supplementation in combating aging-induced myopathy changes in the rat gastrocnemius muscle via enhancement of myogenic stellate cells.

Our data showed that oral probiotics (*Lactobacillus acidophilus*) supplementation at a dose of 1 ml (1 × 109 CFU/ml/day) to rats alleviated gastrocnemius muscle weight and function (evaluated by BIOPAC). In addition, improvements in oxidative stress and muscle satellite cells (CD34 and myogenin) were observed. Finally, the histopathological evaluation confirmed the improvement in aging-induced myopathy changes in rats with oral probiotic supplementation.

The present work reported that the probiotic-treated old group showed a significant increase in the relative muscle weight ratio compared to the probiotic-free old group, which was in agreement with a study performed by Giron et al. [26] which explained this increase by the role of the gut microbiota in increasing the sensitivity of skeletal muscle to anabolic stimuli. Also, probiotics could maintain the anabolism in skeletal muscle via stimulation of protein synthesis and suppression of inflammation [27]. Moreover, Casati et al. [28] have created nutritional regimens that include probiotics to target muscle mass and function.

Concerning the contractile parameters, probiotic-treated old group showed a significant improvement of muscle function compared to probiotic-free old groups. It can be explained by the effect of probiotics in enhancing the proliferation of myogenic stellate cells, resulting in increasing the muscle power. It was previously demonstrated that the elimination of all satellite cells completely prevented muscle hypertrophy because the proliferation of satellite cells could promote the differentiation of muscle fibers which leads to an increase in the muscle bulk [29].

Aging increased the muscle oxidative stress indicated by higher levels of NO and MDA and lower levels of GSH in the old control rats compared to the adult control rats. Probiotics exhibited an antioxidative ability by significantly inverse this change in both the adult + probiotics group and the old + probiotics group. This result was in line with other previous studies, Amaretti et al. [30], Zhang et al. [31], and Wang et al. [32], which demonstrated that the use of probiotics decreases oxidative stress and increases antioxidant capacity. Probiotics may achieve its antioxidant activity via upregulated nuclear factor erythroid 2-related factor 2 (Nrf2) that is a critical regulator against oxidative stress [33].

Regarding the evaluation of muscle satellite cells, our results reported that the probiotics significantly increased the expressions of the myogenin and CD34 levels at both mRNA and protein levels in the old + probiotics and adult + probiotics groups compared to the control group as determined by qRT-PCR and immunohistochemistry, respectively, meaning that the addition of probiotics can improve muscle strength in both physiological and pathological conditions. Myogenin and CD34 are considered markers for satellite cells, which lead to enhanced muscle proliferation. Myogenin is reported to be a key transcription factor for myogenesis, while CD34 plays a vital role in the regulation of lineage-primed progenitor compartments in skeletal muscle [34, 35]***.*** It is well known that muscle regeneration can occur in several stages. In the stationary phase, satellite cells are not functioning. After stimulation of satellite cells, they enter the cell cycle again and proliferate to become myoblasts. After the proliferation phase, myoblasts exit the cell cycle and differentiate into mature muscle cells [36].

Considering the histopathological examination, the old control group showed an apparent increased presentation of the connective tissue spaces of both endo- and perimysia. On the other hand, there was some improvement in the adult + probiotics group in the form of less connective tissue spacing. This finding was in agreement with the results of Fede et al. [37], which reported an increase in the connective tissue spaces in the aged groups. Similarly, Abdel-Halim et al. [38] demonstrated that the disuse atrophy caused by aging or any other cause can lead to muscle atrophy in the form of marked separation of muscle fibers. It could be explained by the increased accumulation of collagen in skeletal muscle and decreased expression of extracellular matrix (ECM) components in aging [39].

Concerning Masson trichrome stain results, the adult control group showed the normal distribution of the bluish-stained collagen fibers. On the contrary, the old control group showed an apparent increased presentation of collagen fibers, in agreement with Guzzoni et al. [40], who reported that bluish-stained collagen fibers increased in the old sedentary group. Moreover, the present study showed that the probiotic supplementation to the adult group produced less marked collagen fibers. It can be explained by decreasing the regenerative potential of the aged skeletal muscle and the loss of function of myogenic progenitor cells. Also, the deposition of ECM in aged skeletal muscles leads to an increase in their stiffness [41].

## Conclusion

In conclusion, our findings confirmed the potential of probiotics attenuate myopathy changes in aging rats. In our study, probiotic supplementation enhanced the weight and contractile properties of rats’ gastrocnemius muscles. This effect is mainly due to the activation of the myogenic stellate cells, myogenin, and CD34 and their antioxidant activity. Probiotic supplementation could be a prospective therapeutic approach to alleviate aging-related myopathy.

## Limitations of the Study

To wholly comprehend how probiotics ameliorated aging-induced myopathic changes in various experimental circumstances, more research is required. Also, evaluation of other myogenic transcription factors could be beneficial.

## Data Availability

The data sets analyzed during the current study are available from the corresponding authors on reasonable request.
